# A Novel Cysteine Protease from *Phytolacca americana* Cleaves Pokeweed Antiviral Protein Generating Bioactive Fragments

**DOI:** 10.3390/plants14152441

**Published:** 2025-08-07

**Authors:** Annabelle Audet, Jennifer A. Chivers, Katalin A. Hudak

**Affiliations:** Department of Biology, York University, Toronto, ON M3J 1P3, Canada; abellea@yorku.ca (A.A.); jchivers@yorku.ca (J.A.C.)

**Keywords:** cysteine protease, apoplast, ribosome-inactivating protein, RNA N-glycosylase, pokeweed antiviral protein

## Abstract

The apoplast is often the first point of contact between plant cells and invading pathogens, serving as an important site for defense signaling. Pokeweed antiviral protein (PAP), a ribosome-inactivating protein from *Phytolacca americana* (pokeweed), is localized to the apoplast and is hypothesized to accompany a pathogen to the cytosol, where it would inactivate host ribosomes to prevent pathogen spread. However, it is not known whether PAP interacts with other proteins in the apoplast. In this study, we identified *Phytolacca americana* cysteine protease 1 (PaCP1), an extracellular cysteine protease, as a novel PAP interactor. Sequence and structural analyses classified PaCP1 as a member of the C1A subfamily of papain-like cysteine proteases. Immunoprecipitation, mass spectrometry, and yeast two-hybrid analysis showed that PAP specifically binds the mature, active form of PaCP1. Curiously, PaCP1 cleaves PAP at its N- and C-termini, generating peptides that enhance MAPK phosphorylation in pokeweed leaves, indicating their potential role in stress signaling. PaCP1 processing of PAP to generate bioactive peptides diversifies the function of a ribosome-inactivating protein beyond its canonical inhibition of translation. Our findings present a novel extracellular role for PAP and advance our understanding of how protein interactions in the apoplast contribute to plant immune responses.

## 1. Introduction

The apoplast is an important extracellular compartment in plants, comprising the cell walls, extracellular spaces, and the xylem. It serves as a dynamic border between the plant and its environment, facilitating nutrient transport, signaling, and defense against pathogens [1,2,3]. During environmental stresses, the apoplast undergoes biochemical changes, such as the accumulation of reactive oxygen species (ROS), antimicrobial compounds, and defense proteins, reinforcing its role as a barrier against pathogen attacks [4,5,6]. The apoplast also hosts a diverse proteome, including enzymes and signaling peptides essential for maintaining cellular homeostasis and coordinating responses to biotic and abiotic stresses [7,8].

Among the apoplastic components, proteases are integral to plant immunity and stress responses. These enzymes catalyze the hydrolysis of peptide bonds, contributing to processes such as protein turnover, defense activation, and programmed cell death [9,10,11,12]. Papain-like cysteine proteases (PLCPs) are a significant class of proteases in the apoplast, many of which facilitate the plant’s immune response. PLCPs activate defense-related proteins, process damage-associated molecular patterns (DAMPs), and degrade pathogen-derived proteins, thereby amplifying immune signaling [12,13]. Their activity is tightly regulated by pro-inhibitory domains and spatial compartmentalization, to prevent unintended proteolysis [6,9,14].

Ribosome inactivating proteins (RIPs) are another component of plant defenses, primarily studied for their role in halting protein synthesis [15,16,17,18]. RIPs are RNA N-glycosylases that hydrolyze a specific adenine base from the conserved α-sarcin-ricin loop of the large ribosomal RNA (rRNA), rendering ribosomes inactive [19,20]. Among well-characterized RIPs, pokeweed antiviral protein (PAP) from *Phytolacca americana* (pokeweed) has been shown to depurinate viral RNAs in addition to rRNA [16,21,22,23]. Interestingly, PAP’s localization to the apoplast [24,25] suggests it may have additional roles beyond its canonical rRNA depurination.

In the current work, we searched for PAP protein interactors within the apoplast. While previous studies identified ribosomal protein L3 and the translation factor eIFiso4G [26,27] as proteins that bind to PAP, little is known about proteins that may regulate PAP. Here, we identify a novel cysteine protease from pokeweed that binds and cleaves PAP within the apoplast. The peptides generated from PAP cleavage enhance MAPK phosphorylation when introduced to leaves, suggesting a previously unrecognized signaling function for PAP in the extracellular space. These findings expand our understanding of RIPs, highlighting a potential role that extends beyond their enzymatic activity in damaging rRNA.

## 2. Results

### 2.1. Identification of PAP Interactors

To identify proteins interacting with PAP in pokeweed, we performed immunoprecipitation assays using a PAP-specific antibody followed by mass spectrometry. As a negative control, we used a FLAG antibody in the same assay, as a pokeweed knockout for PAP was not available to us. To minimize false positives, we considered only proteins detected in at least three biological replicates as high-confidence interactors, with a false discovery rate (FDR) of <0.01 at both the peptide and protein levels. Our analysis resulted in the identification of six PAP-interacting proteins. We inferred the identity, subcellular localization, and biological function of these proteins through BLASTp searches of their amino acid sequences (Supplementary Table S1). Among the interactors, we prioritized further investigation of a protein encoded by pokeweed gene ID *anno1.g16742* [28], given its predicted localization to the apoplast.

**Figure 1:**
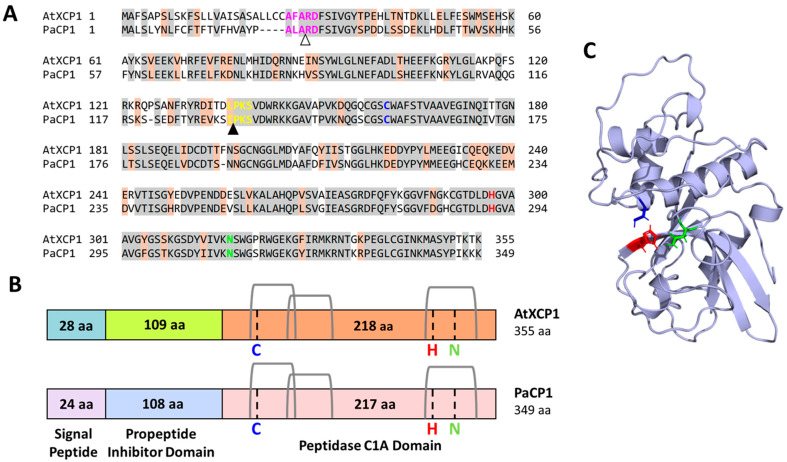
Protein sequence characterization of PaCP1. (**A**) The amino acid sequence for PaCP1 was aligned to the plant protein sequence with the highest homology, AtXCP1, using Blastp. Identical residues are shown in gray highlights and orange highlights indicate residues with similar chemical properties. Pink residues are required for recognition and cleavage of the signal peptide, and this cleavage site is indicated by the white arrow. Yellow residues are required for the recognition and cleavage of the propeptide inhibitor domain, and this cleavage site is indicated by the black arrow. The residues of the active site are colored in blue, red, and green and represent cysteine (C156), histidine (H291), and asparagine (N311), respectively. (**B**) Comparison schematic of the putative protein sequence organization for PaCP1 compared to the known protein organization of AtXCP1, obtained from UniProt (uniprot.org). The location of the signal peptide, propeptide inhibitor domain and peptidase C1A domain for PaCP1 were predicted using Interproscan, Signal-P 6.0, and Target-P 2.0. The active site residues for catalytic activity are cysteine (C; blue), histidine (H; red), and asparagine (N; green). Three disulfide bridges are shown in gray. aa: amino acids. (**C**) Structural prediction of the mature PaCP1 protein (peptidase C1A domain, residues 133–345) using the colab implementation of AlphaFold2. The three catalytic residues of the active site are colored as presented in (**A**,**B**).

A Blastp search against the Embryophyta clade in the SWISS-PROT database classified this PAP interactor as a putative cysteine protease, with *Arabidopsis thaliana* xylem cysteine protease 1 (AtXCP1; TAIR reference ID: AT4G35350) being the closest homolog (Figure 1A) (blast.ncbi.nlm.nih.gov; accessed on 14 May 2024). Global alignment revealed 69% amino acid sequence identity (244/355 residues) between the pokeweed protein and AtXCP1, supporting its annotation as *P. americana* cysteine protease 1 (PaCP1). To further characterize PaCP1, an InterProScan search identified conserved regions belonging to the papain-like cysteine peptidase superfamily, specifically subfamily C1A in accordance with the MEROPS database [29]. The active site residues (C156, H291, and N311) formed the catalytic triad, a characteristic of cysteine proteases [10,14]. Domain analysis identified residues 1–24 as a signal peptide, residues 25–132 as a protease inhibitor domain, and residues 133–345 as the peptidase C1A domain (Figure 1B). Signal-P 6.0 and Target-P 2.0 predicted the cleavage of the signal peptide at residue 24, and the inhibitor domain cleavage site was inferred from sequence homology with AtXCP1 (Figure 1A, arrows). DeepLoc 2.0 further predicted its localization to the extracellular space with high confidence (0.7699). Structural modeling using AlphaFold2 provided a high-confidence structure for the mature PaCP1 peptidase C1A domain (Figure 1C). Together, these bioinformatic and structural analyses indicate that PaCP1 is a PLCP with conserved features typical of this enzyme class.

### 2.2. Characterization of PaCP1 Enzyme Activity

**Figure 2:**
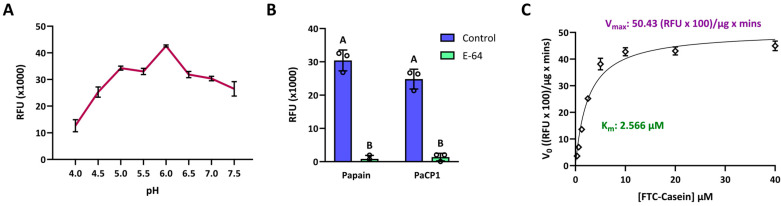
Enzymatic activity of recombinant PaCP1. (**A**) Proteolytic activity of PaCP1 over a range of pH (4–7.5). RFU of the cleaved fluorescent substrate was measured after a 3 h incubation with the protease at 22 °C. Values are means ± standard deviation of n = 3 independent experiments. (**B**) Proteolytic activity of papain and PaCP1. Control samples represent an incubation with the fluorescent substrate and papain or PaCP1 only. The E-64 samples are supplemented with 100 μM E-64 specific inhibitor. Values are means ± standard deviation of n = 3 independent experiments. Different letters above the bars represent statistically significant differences. *p* values were calculated by two-way ANOVA (*p* < 0.001). (**C**) Enzyme kinetics of PaCP1 (0.1 µg) at varying FTC-Casein substrate concentrations (0.3 µM to 40.0 µM) after a 1 h incubation at 22 °C. Values are means ± standard deviation of n = 3 independent experiments.

To confirm PaCP1’s identity as a cysteine protease and assess its enzyme activity, we performed proteolytic activity assays using recombinant PaCP1. The mature form of PaCP1, lacking the signal sequence and protease inhibitor domain, was expressed in *Escherichia coli* with a C-terminal 6x His tag and purified via affinity chromatography. Enzymatic activity was evaluated using FTC-casein as a fluorogenic substrate, with fluorescence intensity measured in relative fluorescence units (RFU). PaCP1 exhibited optimal activity at pH 6 (Figure 2A) and this pH was used for all further analyses. PaCP1’s enzyme activity was comparable to that of papain, which served as the model protease, and significantly reduced by the inhibitor E-64, confirming PaCP1’s classification as a cysteine protease (Figure 2B). Kinetic assays revealed a K_m_ of 2.566 µM and a V_max_ of 5043 RFU/µg × min, indicating high substrate affinity (Figure 2C). These results establish PaCP1 as a functional cysteine protease and provide insights into its enzymatic properties.

### 2.3. Support for PAP-PaCP1 Interaction

**Figure 3:**
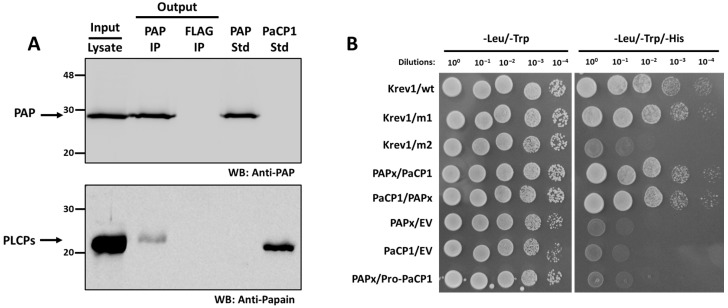
Support for PAP-PaCP1 interaction. (**A**) Immunoblot of the immunoprecipitation assay using PAP to immunoprecipitate PLCPs in pokeweed. A FLAG immunoprecipitation serves as a negative control. The input lane represents total pokeweed protein from cell lysate. The PAP and FLAG output lanes represent the immunoprecipitated proteins. A PAP and PaCP1 standards are included. Proteins were detected by Western blot (WB) using anti-PAP and anti-papain antibodies. (**B**) Yeast-two hybrid assay testing the interaction between PAPx and PaCP1. Growth of yeast cells on -Leu/-Trp/-His media represent positive protein–protein interaction. The same cells were plated on -Leu/-Trp to confirm yeast transformation. Yeast transformed with empty vectors (EVs) served as negative activation control. Pro-PaCP1 is PaCP1 with its pro-inhibitory domain. Krev1/wt: strong positive interaction control. Krev1/m1: weak positive interaction control. Krev1/m2: negative interaction control.

We tested the interaction between PaCP1 and PAP by Western blot analysis of the PAP immunoprecipitation samples. Total protein from pokeweed cell lysate indicated the presence of PAP and a PLCP (Figure 3A). Using a PAP-specific antibody, we immunoprecipitated PAP with a PLCP and confirmed both proteins using respective antibodies (Figure 3A). Since pokeweed is not amenable to genetic transformation and deletion mutants are unavailable, we included a negative control immunoprecipitation using a FLAG antibody. In this control, neither PAP nor PLCPs were detected (Figure 3A). As a PaCP1-specific antibody was not available, we used a papain-specific antibody, which was validated by the presence of a band in the lane containing purified PaCP1 standard (Figure 3A; PaCP1 std). These findings support the interaction between PAP and a papain-like cysteine protease in pokeweed.

To determine whether PAP bound to the mature or precursor form of PaCP1, we performed a yeast two-hybrid assay. The mature PaCP1 sequence (C1A peptidase domain only) or its precursor Pro-PaCP1 (pro-inhibitory domain plus C1A peptidase domain) and PAPx (an inactive mutant of PAP) [30] were cloned into bait and prey vectors. PAPx was used to avoid inhibition of yeast growth associated with rRNA depurination by native PAP. Yeast transformed with constructs encoding mature PaCP1 and PAPx exhibited growth on -Leu/-Trp/-His selective media, confirming their interaction (Figure 3B). Notably, no interaction was observed between PAPx and Pro-PaCP1 (pro-inhibitory domain plus C1A peptidase domain), indicating that PAPx specifically interacted with the mature, active form of PaCP1 (Figure 3B). Control experiments with empty vectors, self-activation controls, and interaction-deficient mutants validated the assay’s specificity.

### 2.4. Co-Localization of PAP and PaCP1

**Figure 4:**
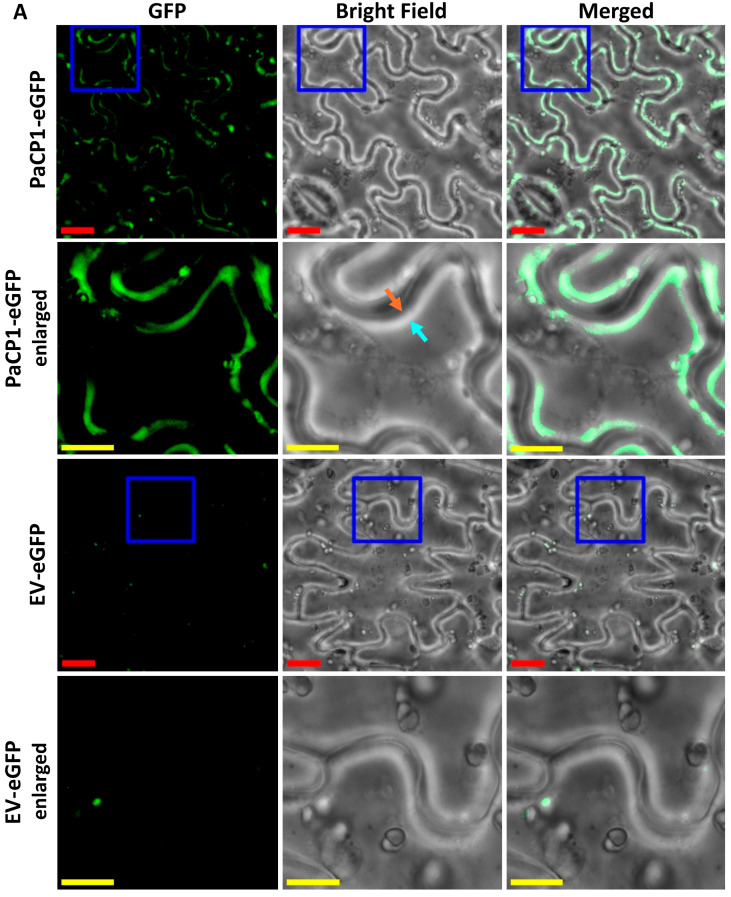

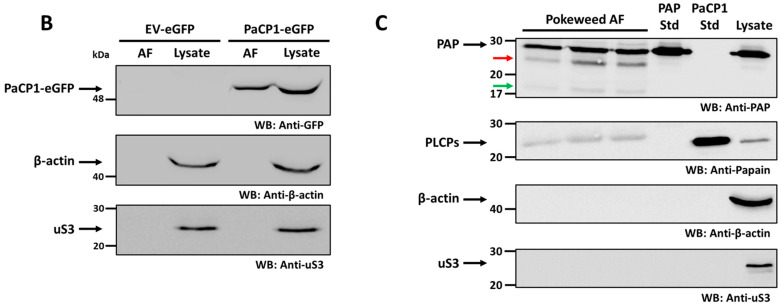
PaCP1 and PAP localization to the extracellular space. (**A**) Fluorescence microscopy images showing PaCP1-eGFP localized to the extracellular space of *N. benthamiana* leaf epidermal cells, after salt-induced plasmolysis. EV-eGFP served as a negative control. Enlarged regions of interest (blue square) are shown below each original image. Red scale bars = 20 μM; yellow scale bars = 5 μM. Orange arrow indicates the plant cell wall, and the cyan arrow indicates the plasma membrane pulling away from the cell wall. (**B**) Western blot of apoplastic fluid (AF) and cell lysates from *N. benthamiana* leaves expressing PaCP1-eGFP or EV-eGFP, probed with an anti-GFP antibody. WB using anti-β-actin and anti-ribosomal protein uS3 antibodies served as controls for the absence of cytoplasmic protein contamination. (**C**) Western blot using anti-PAP and anti-papain antibodies to confirm presence of PAP and papain-like cysteine proteases in apoplastic fluid extracted from pokeweed leaves. Three samples of independent apoplastic fluid extractions from pokeweed are shown. The red and green arrows represent the lower molecular weight cleavage products of PAP. PAP and PaCP1 protein standards served as positive and/or negative controls. A sample of total pokeweed proteins was loaded in the far-right lane.

To investigate the cellular localization of PaCP1, we expressed full-length PaCP1 (signal peptide, pro-inhibitory domain and peptidase domain) fused with eGFP in *Nicotiana benthamiana* via agroinfiltration. We chose *N. benthamiana* as pokeweed is not amenable to *Agrobacterium tumefaciens*-mediated transformation. Fluorescence microscopy of tobacco leaf epidermal cells revealed PaCP1-eGFP localized to the extracellular space upon plasmolysis, whereas empty vector control (EV-eGFP) showed little eGFP (Figure 4A). To better illustrate localization and plasmolysis, we included an enlarged region of interest for both PaCP1-eGFP and EV-eGFP images in Figure 4A. The orange arrow indicates the cytoplasmic face of the cell wall whereas the cyan arrow points to the plasma membrane pulled away from the cell wall. The white band between these arrows in the brightfield image indicates the extracellular space upon plasmolysis and overlaps with the location of eGFP expression in the merged image. To further support the apoplastic localization of PaCP1, we extracted apoplastic fluid (AF) from these infiltrated tobacco plants and performed Western blot using an anti-GFP antibody. Our results confirmed the presence of the PaCP1-eGFP fusion protein in apoplastic fluid and its absence in the EV-eGFP control (Figure 4B). As an additional control, Western blot for the cytoplasmic markers β-actin and ribosomal protein uS3 confirmed their presence in total cell lysates but absence in apoplastic fluid, ruling out cytoplasmic contamination (Figure 4B). To extend this finding to pokeweed, we extracted apoplastic fluid from pokeweed leaves and detected papain-like cysteine proteases using an anti-papain antibody (Figure 4C). Since a PaCP1-specific antibody was unavailable, our approach did not definitively confirm the presence of PaCP1 but did indicate PLCPs in pokeweed apoplastic fluid. Additionally, PAP was present in apoplastic fluid samples along with visible lower molecular weight fragments (Figure 4C red and green arrows). Collectively, these results support the extracellular localization of both PaCP1 and PAP in pokeweed.

### 2.5. PaCP1-Mediated Cleavage of PAP

**Figure 5:**
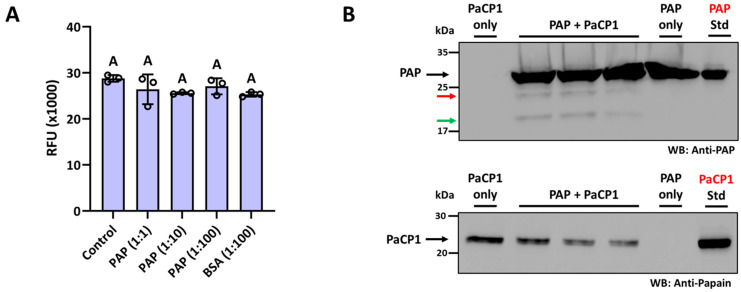
PaCP1 cleaves PAP. (**A**) Enzymatic activity of recombinant PaCP1 in the presence of increasing amounts of PAP. Fluorescent substrate and PaCP1 were either alone (control) or were supplemented with increasing amounts of PAP (1:1, 1:10 and 1:100; PaCP1: PAP). BSA served as a negative control. RFU of the cleaved fluorescent substrate was measured after a 3 h incubation at 22 °C. Values are means ± standard deviation of n = 3 independent experiments. Different letters above the bars represent statistically significant differences. *p* values were calculated by one-way ANOVA (*p* > 0.05). (**B**) Western blot using anti-PAP showing PAP cleavage after 3 h incubation of PAP (10 μg) with 0.1 μg PaCP1 at 22 °C (three samples are shown). The red and green arrows represent the 24 kDa and 18 kDa cleavage products, respectively. Western blot using anti-papain antibody to confirm the presence of PaCP1 in the samples. PaCP1 alone and PAP alone were negative controls. A PAP and PaCP1 standard as a positive control in the far-right lanes.

The biological significance of the PAP-PaCP1 interaction was investigated by first testing if the binding could affect PaCP1 proteolytic activity. We performed enzymatic cleavage assays with varying PaCP1:PAP ratios (1:1 to 1:100) and did not detect significant changes in PaCP1 proteolytic activity in the presence of all concentrations of PAP (Figure 5A). Buffer only and bovine serum albumin (BSA) served as negative controls. Conversely, we evaluated whether PaCP1 cleaved PAP by incubating proteins together at a 1:100 ratio (PaCP1:PAP) followed by immunoblot analysis with an anti-PAP antibody. Figure 5B illustrates cleavage products of PAP (approximately 24 kDa and 18 kDa) distinct from the full-length 29 kDa protein following incubation with PaCP1. A Western blot using an anti-papain antibody confirmed the presence of purified PaCP1 in these samples. The size of these products matched those observed in the apoplastic fluid samples (Figure 4B), suggesting that PaCP1 cleaves PAP within the apoplast.

### 2.6. Analysis of PAP Cleavage Products

**Figure 6:**
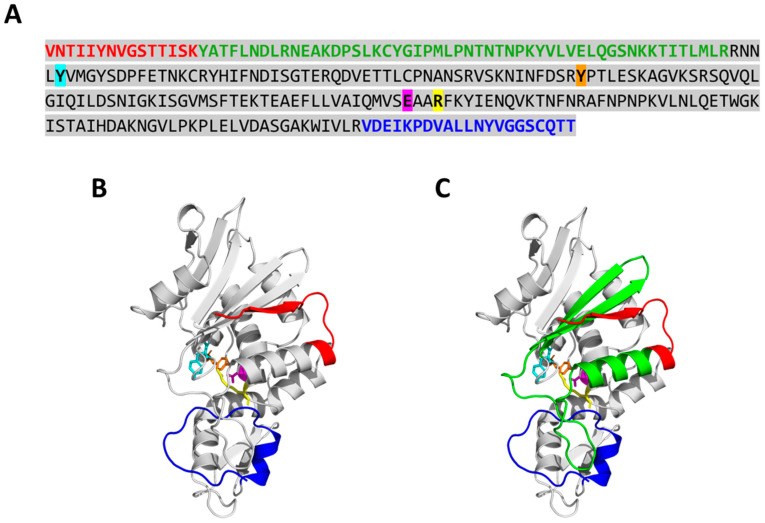
Mass spectrometry analysis of PAP cleavage products. (**A**) Amino acid sequence of the 24 kDa and 18 kDa PAP cleavage products as identified by mass spectrometry. The amino acids colored in red (N-terminus) and blue (C-terminus) were missing from the 24 kDa PAP product. The amino acids in red and green (N-terminus) and blue (C-terminus) were missing from the 18 kDa PAP product. The amino acid sequence of mature full-length PAP is highlighted in gray. The amino acids of the PAP active site are colored as follows: tyrosine (Y72) in cyan, tyrosine (Y123) in orange, glutamic acid (E176) in pink, and arginine (R179) in yellow. (**B**,**C**) Structure of PAP obtained from the AlphaFold Protein Structure Database (AF-P10297-F1-v4) illustrating missing regions for the 24 (red and blue) and 18 kDa (red, green, and blue) product, respectively. The amino acids are colored the same as in (**A**).

To identify the regions of PAP cleaved by PaCP1, mass spectrometry analysis was performed on the 24 kDa and 18 kDa PAP cleavage products extracted from SDS-PAGE gels. The peptides identified aligned to the PAP sequence, revealing that both products lacked amino acids from their N- and C-termini (Figure 6A). The 24 kDa product was missing 15 N-terminal and 26 C-terminal residues, while the 18 kDa product lacked 67 N-terminal and the same 26 C-terminal residues. Structural mapping indicated that the cleavages removed specific secondary structural elements, including an alpha helix and two beta sheets from the 18 kDa product compared to the 24 kDa product (Figure 6B,C). Despite these cleavages, PAP’s active site remained intact. These findings suggest that PaCP1 selectively cleaves PAP’s terminal regions, potentially modulating its function.

**Figure 7:**
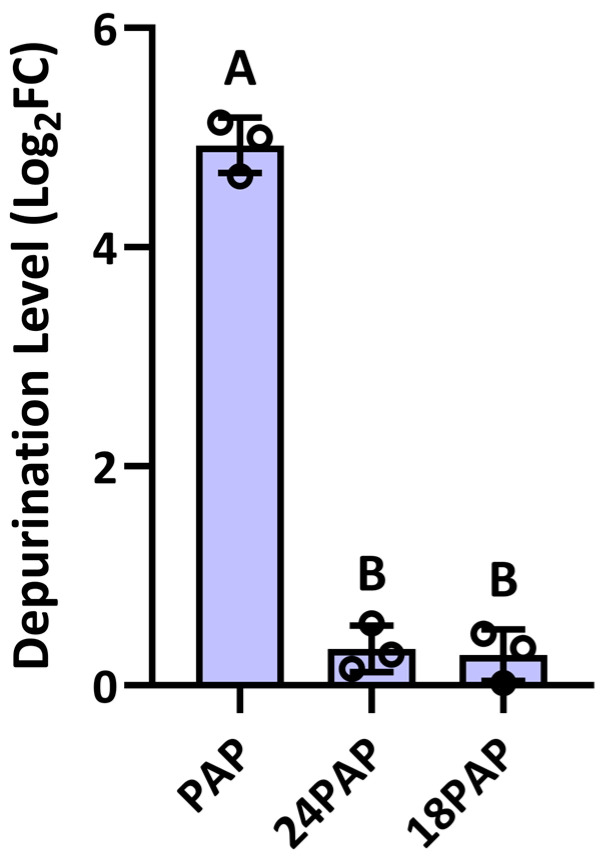
Depurination activity of PAP cleavage products. qRT-PCR analysis of the levels of rRNA depurination of the 24 (24PAP) and 18 (18PAP) kDa PAP products. Purified *N. benthamiana* ribosomes (50 µg) were incubated with either 24PAP or 18PAP (5 µg), or full-length PAP for 30 min at 30 °C followed by rRNA isolation. qRT-PCR measured the levels of reference and target 25S rRNA products relative to samples treated with buffer alone. Different letters above the bars represent statistically significant differences. Values are means ± standard deviation of n = 3 independent experiments. *p* values were calculated using one-way ANOVA (*p* <0.01).

To assess the depurination activity of PAP cleavage products, we purified recombinant 24 kDa and 18 kDa PAP fragments expressed in *E. coli*. These proteins were incubated with ribosomes extracted from *N. benthamiana*, and a qPCR-based depurination assay was performed to evaluate their activity. Full-length PAP incubated with ribosomes served as a positive control for rRNA depurination. The assay revealed that both the 24 kDa (24PAP) and 18 kDa (18PAP) PAP cleavage products exhibited significantly lower depurination activity compared with full-length PAP (Figure 7). Therefore, proteolytic cleavage of PAP substantially reduced its enzyme activity, impacting its biological function as an RNA N-glycosylase.

### 2.7. Activation of MAPK Signaling by the Cleaved Peptides

**Figure 8:**
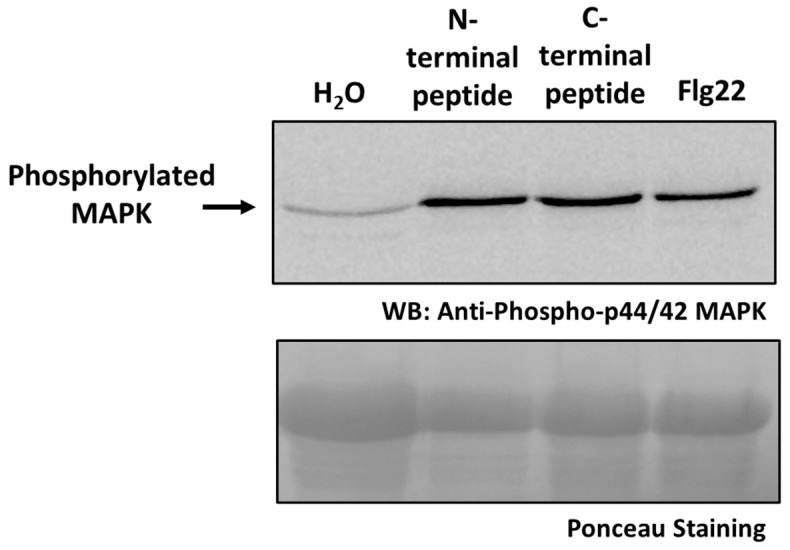
Increase in MAPK phosphorylation by N- and C-terminal peptides of PAP. Immunoblot using anti-phospho p44/42 MAPK polyclonal antibody showing MAPK phosphorylation in protein lysates (35 μg) from pokeweed leaves infiltrated with either N-terminal or C-terminal peptide from the 24 kDa PAP cleavage product. Leaves infiltrated with water or Flg22 served as negative and positive controls, respectively. The blot was stained with Ponceau to illustrate loading amounts.

To further investigate the biological significance of PaCP1-mediated cleavage of PAP, we evaluated whether the small peptides released from the N- and C-termini of PAP to generate the 24 kDa cleavage product influenced plant signaling. Synthetic peptides corresponding to the N- and C-terminal residues were commercially synthesized and infiltrated into pokeweed leaves, along with water as a negative control and the bacterial flagellin-derived peptide Flg22 as a positive control. To assess the activation of immune signaling, we performed immunoblot analysis using an antibody specific to phosphorylated mitogen-activated protein kinases (MAPKs). A single phosphorylated MAPK band was detected, consistent with the activation of the MPK6 homolog in pokeweed, given its persistent phosphorylation 24 h following treatment (Figure 8). Both the N- and C-terminal peptides triggered a significant increase in MAPK phosphorylation levels, comparable to the response elicited by Flg22 (Figure 8). These findings suggest that the peptides derived from PAP cleavage may act as signaling molecules in the apoplast, potentially playing a role in plant immune responses.

## 3. Discussion

In this study, we identified a novel interaction between PAP and PaCP1, a newly identified PLCP in pokeweed. We characterized PaCP1 as a cysteine protease with optimal activity at pH 6 and a proteolytic efficiency comparable to papain, a known cysteine protease. Both PAP and PaCP1 co-localized to the apoplast, where PaCP1 selectively cleaved PAP at its N- and C-termini, resulting in 24 and 18 kDa fragments that lacked ribosomal depurination activity. However, the small peptides generated by PaCP1-mediated cleavage of PAP activated the MAPK signaling pathway in pokeweed leaves, suggesting an immune-related role for PAP in the extracellular space. Our findings show a novel function for PAP in the apoplast, extending its significance beyond its well-known rRNA depurination activity.

While the natural substrates of PaCP1 remain to be fully characterized, our results confirm that PAP is a substrate under physiological conditions. PaCP1 likely exhibits broad substrate specificity, as PLCPs generally lack strict sequence recognition motifs and instead rely on local structural features and accessible peptide bonds for substrate selection [9,10,11,12,13,14]. The high affinity of PaCP1 for PAP, reflected in its low K_m_ value, suggests efficient substrate recognition. These features indicate that PaCP1 is an active apoplastic protease possibly capable of targeting a range of extracellular proteins.

PAP is a well characterized RNA N-glycosylase that hydrolyzes a purine base from rRNA [19,20]. While its accumulation in the apoplast may protect plant ribosomes from depurination, it also positions PAP in a compartment important for the plant’s immune response. We show that PaCP1 localized to the apoplast, and that PAP interacted only with the mature PaCP1 (peptidase domain only), producing PAP fragments that were also found in the apoplast. Taken together, we suggest that these fragments are generated when both proteins reach the extracellular space, indicating that active PaCP1 must be present in the apoplast. Since the apoplast is the primary location of pathogen detection and defense signaling [2,3,4,31,32], the interaction between PAP and PaCP1 supports a new function for PAP based on its extracellular localization.

Our findings show that PaCP1 cleaves PAP at its N- and C-termini, producing 24 and 18 kDa fragments that do not retain depurination ability. This loss of activity is consistent with previous work showing that deleting 16 N-terminal or 19 C-terminal amino acids from PAP eliminates its depurination activity and cytotoxic effects in yeast [32]. In line with these observations, the 24 kDa fragment, which is missing the first 15 N-terminal residues, and the 18 kDa fragment which is missing the first 67 N-terminal residues failed to depurinate ribosomes. Additionally, the absence of the final 26 C-terminal amino acids in both fragments also likely explains the loss of enzymatic activity. Since this cleavage occurs in the apoplast, where ribosomes are absent, the lack of rRNA depurination activity presumably does not impact PAP’s extracellular function. Rather, the PAP fragments could serve non-enzymatic roles, such as binding extracellular RNAs. Recent studies have shown the presence of extracellular RNAs outside vesicles in both animals and plants [33,34,35,36]. If the PAP fragments retain their ability to bind RNA, despite losing enzymatic activity, they could protect these RNAs from degradation by apoplastic nucleases. Moreover, the partial proteolysis of PAP by PaCP1, shown by significant amounts of intact PAP remaining in apoplastic fluid (Figure 4B and Figure 5B), suggests that these fragments most likely complement rather than replace the functions of full-length PAP.

Although only a small fraction of PAP is cleaved by PaCP1 under normal conditions, this cleavage may increase during pathogen attack, facilitated either by enhanced transcription or activation of PaCP1. Based on our previous transcriptome analysis of pokeweed following various stress treatments, we noted that transcript levels of PaCP1 significantly increased following treatment with jasmonic acid, as did transcript levels of PAP [37]. Jasmonic acid mediates the plant response to herbivores and necrotrophic pathogens [38,39]; therefore, elevated expression of PaCP1 and PAP following JA treatment suggests roles for each enzyme which may involve increased cleavage of PAP and release of bioactive peptides involved in defense. In addition to changes in transcript levels, stress may enhance activity of PaCP1. For example, stress-induced alkalinization of the apoplast, documented in other plants [40,41,42,43], is hypothesized to occur in pokeweed as well, potentially enhancing PaCP1 activity and promoting PAP cleavage. The pH preference of PaCP1 aligns with those of other cysteine proteases, such as AtXCP1, which also exhibits optimal activity at pH 6 and contributes to plant immune signaling by activating defense-related peptide AtCAPE9 from PR1 [44]. If a similar stress-induced alkalinization occurs in pokeweed, it could create conditions favorable for PaCP1 activity, possibly enhancing immune signaling through the increase in PAP cleavage and the release of the peptides. In maize, for example, apoplastic PLCPs cleave the propeptide precursor ProZip1 to release Zip1, a damage-associated molecular pattern (DAMP) that amplifies immune responses [45]. Zip1 enhances salicylic acid accumulation, which upregulates PLCP activity in a positive feedback loop, and activates defense-related genes. Similarly, in wheat, the PLCP TaRD21A was shown to cleave PROWIP1 into Wip1, an immune signaling peptide that reduces viral infection [46]. These examples highlight the ability of PLCPs to release immune-regulating peptides. In pokeweed, the cleavage of PAP by PaCP1 may represent a comparable mechanism for activating immune signaling.

The role of PAP-derived peptides remains speculative; however, our data show that these peptides increase MAPK phosphorylation, which likely activates MAPK pathways. Whether these peptides directly lead to MAPK activation or function within a broader defense-related process is unclear. Although both MPK3 and MPK6 may be phosphorylated in response to treatment, previous studies have shown that MPK3 phosphorylation often diminishes by later time points, while MPK6 remains persistently activated [47,48,49,50,51]. Since we extracted protein at 24 h post-treatment, the observed band likely represents a dominant MPK6-like homolog in pokeweed. Plant elicitor peptides, such as systemin in tomato, AtPep1 in *Arabidopsis*, and ZmPep1 in maize, are known to enhance immune responses against bacteria, herbivores, and fungi [52,53,54,55,56,57]. These peptides are typically derived from precursor proteins that accumulate and are processed under specific stress conditions [58,59]. While PAP does not fit the traditional profile of a precursor protein, the possibility that PAP-derived peptides interact with pattern-recognition receptors (PRRs) resulting in the activation of MAPK pathways is consistent with the role of plant elicitor peptides in immune signaling.

To our knowledge, this is the first report of a ribosome inactivating protein being cleaved by a host protease to generate bioactive products. Based on these findings, we propose that PAP has a dual function: an early role in immune signaling via PaCP1-mediated cleavage in the apoplast, followed by a cytotoxic role involving ribosome depurination if the immune response becomes overwhelmed. This two-tiered response may also depend on the type of stress experienced and may assist the plant to balance defense with self-preservation.

## 4. Materials and Methods

### 4.1. Plant Materials and Growth Conditions

Pokeweed seeds were treated with sulfuric acid for 5 min, rinsed, and hydrated in water for 5–7 days until their seed coats cracked. Germinating seeds were sowed in PromixBx soil mix and placed on heat mats. *N. benthamiana* seeds were sown in the same soil mix. Both species were cultivated in growth chambers (AC60, Biochambers, Winnipeg, MB, Canada) with a 14 h light/10 h dark cycle at 24 °C (light) and 21 °C (dark) under fluorescent and incandescent light at 180 µE m^−2^ s^−1^. Plants were watered every day and fertilized once every two weeks with N:P:K 20:20:20 fertilizer. Leaf tissues were harvested at the 6 to 8 leaf stage, midveins removed, flash-frozen in liquid nitrogen, and stored at −40 °C.

### 4.2. Total Protein Isolation

Pokeweed and tobacco leaf tissue (approximately 200 mg) was ground in liquid nitrogen and suspended in 400 µL of cold protein extraction buffer (50 mM Tris-HCl pH 7.5, 1 mM EGTA, 1 mM DTT, 1x protease inhibitor (Thermo Fisher Scientific, Whitby, ON, Canada), 5% glycerol). The samples were centrifuged at 10,000× *g* for 5 min at 4 °C to remove cellular debris. The supernatants were collected, and total protein concentration was determined by Bradford assay. Proteins to be used for immunoblotting assays were mixed 1:1 with 2× Laemmli buffer and heat denatured at 95 °C.

### 4.3. Immunoprecipitation Assay and Mass Spectrometry

Anti-PAP and anti-FLAG (to serve as negative control, Millipore Sigma, Oakville, ON, Canada) antibodies were crosslinked to Protein A magnetic beads (New England Biolabs, Whitby, ON, Canada). Immunoprecipitation assays were performed using 1 mg of total pokeweed protein and 40 µL of antibody-bead mix with additional TBS (50 mM Tris-HCl pH 7.5, 150 mM NaCl) to 1 mL. The samples were incubated with rotation at 4 °C for 1 h. Following incubation, the beads were washed three times with TBS and co-immunoprecipitated proteins were eluted in 30 µL 2× Laemmli buffer. The immunoprecipitated proteins were loaded onto 12% SDS-PAGE, separated for 5 min, and stained with Coomassie Brilliant blue. The bands were excised from the gel and subjected to an overnight trypsin digestion. Peptides were analyzed by the Centre for Research in Mass Spectrometry, York University, Toronto, ON, Canada.

### 4.4. Bioinformatic and Structural Analyses of PaCP1

The pokeweed protein with gene ID anno1.g16742 identified as a PAP interactor by mass spectrometry was analyzed for homology using BLASTp against the Embryophyta clade in the SWISS-PROT database. Proteins with E-values below 1 × 10^−4^ were considered significant, and the top hit was used to annotate the protein. Subcellular localization and functional predictions were based on homologous protein annotations in UNIPROT [60]. Protein domains, superfamily classification, and active sites of PaCP1 were identified using InterProScan (v. 5.67) [61]. Signal peptides were confirmed using Signal-P (v. 6.0) [62] and Target-P (v. 2.0) [63], while subcellular localization was predicted with DeepLoc (v. 2.0) [64]. Global alignment of PaCP1 and AtXCP1 was performed with BLASTp.

Structural prediction for PaCP1 was generated using AlphaFold2, via the open source colab implementation with default settings [65,66]. The PAP structural model was obtained from the AlphaFold Protein Structure Database (AF-P10297-F1-v4) [67]. The resulting models were visualized in PyMOL (v.3.1) to highlight domains and active sites.

### 4.5. Plasmid Construction

Total RNA from pokeweed leaf tissue was isolated using the Monarch Total RNA Miniprep Kit (New England Biolabs, Whitby, ON, Canada) according to the manufacturer’s instructions. For the generation of cDNAs, 500 ng of total RNA was combined with a gene-specific reverse primer, denatured, and reverse-transcribed using Mashup reverse transcriptase [68].

For the generation of recombinant PaCP1, PCR amplification of mature PaCP1 (peptidase domain only) cDNA was performed using Q5 High-Fidelity DNA polymerase (New England Biolabs, Whitby, ON, Canada) with primers introducing restriction enzyme sites for ligation into the pET28a vector (Millipore Sigma, Oakville, ON, Canada). All primers used for cloning are listed in Supplementary Table S2. Amplified PCR products were purified via phenol-chloroform extraction and ethanol precipitation, digested with EcoRI and BlpI, and resolved on low-melt agarose gels. Purified inserts were ligated into digested pET28a vector using T4 DNA ligase (New England Biolabs, Whitby, ON, Canada) and transformed into DH5α *E. coli* for plasmid amplification.

The Gateway™ cloning system (Thermo Fisher Scientific, Whitby, ON, Canada) was used to generate the plasmids for the yeast two-hybrid assay and the subcellular localization assay according to the manufacturer’s instructions. The coding sequences of full-length PaCP1 (signal peptide, pro-inhibitory domain and peptidase domain), Pro-PaCP1 (pro-inhibitory domain and peptidase domain only), mature PaCP1 (peptidase domain only), and PAPx (inactive mutant of PAP with a single amino acid mutation in its active site) [30] were amplified using a two PCR reaction method to introduce the attB1 and attB2 recombination sites at the 5′ and 3′ end, respectively. The first PCR reactions used primers with half of the attB sequences along with gene-specific sequences, and the second PCR reaction used primers with the second half of the attB sequences. PCR reactions were conducted using Q5 High-Fidelity DNA polymerase (New England Biolabs, Whitby, ON, Canada). The attB-flanked DNA fragments were purified using the EZ-10 Spin Column DNA Cleanup Miniprep Kit (Bio Basic, Markham, ON, Canada). The purified fragments were cloned into pDONR221™ (Thermo Fisher Scientific, Whitby, ON, Canada) using BP Clonase™ (Thermo Fisher Scientific, Whitby, ON, Canada) according to the manufacturer’s protocol, thereby generating entry clones. Correct insertion of all coding sequences was verified by Oxford Nanopore Technologies sequencing (Plasmidsaurus, Louisville, KY, USA).

### 4.6. Isolation of His-Tagged PaCP1

The generated pET28a PaCP1 plasmid was expressed in LOBSTR *E. coli* cells. Cultures were grown in LB medium supplemented with chloramphenicol (35 µg/mL) and kanamycin (50 µg/mL) until the optical density (OD_600nm_) reached 0.8. Protein expression was induced with the addition of 0.6 mM IPTG, and cell incubation at 18 °C for 16 h. Cells were harvested and lysed by sonication in lysis buffer (50 mM Tris pH 8.0, 300 mM NaCl, 5% glycerol), and the lysate was cleared by centrifugation. Soluble proteins were purified using nickel affinity chromatography (GE Healthcare, Mississauga, ON, Canada), with sequential imidazole washes and elution. Eluted protein was concentrated and buffer-exchanged using Amicon Ultra-15 centrifugal filter unit concentrator (Millipore Sigma, Oakville, ON, Canada). The quality of purified PaCP1 was assessed by SDS-PAGE alongside BSA standards. Purified protein was stored in protein storage buffer (20 mM Tris-HCl pH 8.0, 1 mM EDTA, 1 mM DTT, 100 mM NH_4_Cl, 20% glycerol) at −80 °C.

### 4.7. In Vitro Enzyme Activity Assay

The enzymatic activity of PaCP1 was assessed through fluorescent protease assays using FTC-Casein (Thermo Fisher Scientific, Whitby, ON, Canada) as a substrate. FTC-Casein is casein labeled with fluorescein isothiocyanate (FITC) which will generate a quantifiable change in fluorescence upon proteolytic cleavage. To determine the optimal pH for PaCP1 activity, reactions were conducted in 0.1 M sodium citrate buffer (pH 4.0 to 6.0) or 50 mM MOPS buffer (pH 6.5 to 7.5). Purified PaCP1 was diluted to a working concentration of 1 µg/mL, mixed with 10 µg/mL of FTC-Casein in 96-well plates (Millipore Sigma, Oakville, ON, Canada), and incubated in the dark for 3 h at room temperature. Fluorescence values were measured using a microplate fluorometer at 485/538 nm excitation/emission and relative fluorescence units (RFU) were calculated by subtracting blank control values. Each experiment was repeated three times with similar results.

Inhibition assays were performed at pH 6.0 with either 1 µg/mL of PaCP1 or papain (Millipore Sigma, Oakville, ON, Canada). Both enzymes were pre-incubated for 30 min with 100 µM E-64 before adding 10 µg/mL of FTC-Casein. Additionally, 1 µg/mL of PaCP1 was pre-incubated for 30 min with varying concentrations of PAP (1 µg/mL, 10 µg/mL, and 100 µg/mL), corresponding to 1:1, 1:10, and 1:100 ratios of PaCP1 to PAP. Pre-incubation with 100 µg/mL of BSA was used as a negative control. Following pre-treatments all reactions were incubated in the dark for 3 h at room temperature. Each experiment was repeated three times with similar results. Enzyme kinetics were analyzed using 0.1 µg of purified PaCP1 and varying concentrations of FTC-Casein (0.3125, 0.625, 1.25, 2.5, 5, 10, 20, 40 µM), with fluorescence measured after 1 h at 22 °C. We chose this temperature to measure PaCP1 activity as this is the temperature of pokeweed’s native environment during its growing season and we wanted to mimic these conditions for the enzyme. Graphpad prism v.10 was used to calculate the V_max_ and K_m_ values using the Michaelis-Menten method.

### 4.8. Yeast-Two Hybrid Assay

The coding sequences of mature PaCP1 (peptidase domain only), Pro-PaCP1 and PAPx from pDONR221™ were cloned into pDEST32™ (Thermo Fisher Scientific, Whitby, ON, Canada) and pDEST22™ (Thermo Fisher Scientific, Whitby, ON, Canada) using LR Clonase (Thermo Fisher Scientific, Whitby, ON, Canada) according to the manufacturer’s protocol, generating the destination clones. The destination clones were transformed into the *Saccharomyces cerevisiae* (yeast) strain MaV203 using the ProQuest™ Two-Hybrid System (Invitrogen, Burlington, ON, Canada) according to the manufacturer’s protocol. The transformed cells were plated on selective SC media lacking leucine and tryptophan (SC -leu/-trp) and grown at 28 °C for 3 days. To assess protein–protein interaction, yeast cells were incubated in selective media, washed, and resuspended in 0.9% NaCl. Cells were serially diluted and plated on selective SC media lacking tryptophan, leucine and histidine (SC -leu/-trp/-his) and supplemented with 25 mM 3-Amino-1,2,4-triazole (3AT, Millipore Sigma, Oakville, ON, Canada). Plates were incubated at 28 °C for 48 h. The interaction experiments were repeated three times with similar results.

### 4.9. Microscopy

The coding sequence of full-length PaCP1 (signal peptide, pro-inhibitory domain and peptidase domain) previously cloned into pDONR221™ was cloned into pGWB405 [69] using LR Clonase™ (Thermo Fisher Scientific, Whitby, ON, Canada) to generate the PaCP1-eGFP construct. An empty vector-eGFP (EV-eGFP) construct, containing a premature stop codon after the first 63 nucleotides of PaCP1 to remove the toxic *ccdB* gene present in Gateway cloning vectors, was used as a negative control. Because of the stop codon, this EV-eGFP would not express eGFP. The constructs were amplified in DH5α *E. coli*, and extracted plasmid DNA was introduced into *Agrobacterium tumefaciens* AGL1 cells via electroporation. Transformed cells were plated on YEP agar supplemented with carbenicillin (50 μg/mL) and spectinomycin (50 μg/mL) for selection. Agrobacterium cultures carrying PaCP1-eGFP or EV-eGFP constructs were grown overnight and co-cultured with helper component protease (HcPro)-expressing Agrobacterium. Cells were resuspended to an OD_600_ of 0.5 with infiltration solution (10 mM MES-KOH, pH 5.6, 10 mM MgCl_2_, 200 μM acetosyringone) and infiltrated into *N. benthamiana* leaves. After 72 h, the leaves were treated with 0.8 M mannitol to induce plasmolysis and epidermal peels from the abaxial surface of the leaves were collected. eGFP fluorescence was observed using a Zeiss Axio Observer A1 epifluorescence microscope with a 489/509 nm excitation/emission.

### 4.10. Apoplastic Fluid Extraction

Extraction was modified from the previously described syringe infiltration method [70]. Pokeweed or tobacco leaves were cut into pieces, rinsed with deionized water, and blotted dry. The leaf pieces were placed in a 50 mL syringe barrel with 40 mL of apoplastic infiltration buffer (20 mM ascorbic acid, 20 mM NaCl, pH 4.0). After creating a vacuum seal using parafilm, the syringe plunger was slowly pulled back to allow the infiltration buffer to infiltrate the leaves. The process was repeated until the leaves appeared darker and more translucent. The leaves were blotted dry, wrapped in parafilm, and placed in a Miracloth (Millipore Sigma, Oakville, ON, Canada) bag suspended in 15 mL tube. The tubes were centrifuged at 2000× *g* for 25 min at 4 °C to collect the apoplastic fluid. Apoplastic proteins were dissolved in 2× Laemmli buffer.

### 4.11. In Vitro Cleavage Assays

To test if PaCP1 cleaves PAP, 100 ng of mature, purified recombinant PaCP1 (peptidase domain only) was mixed with 10 µg of purified PAP [25] in 0.1 M sodium citrate buffer at pH 6 and incubated at room temperature for 4 h while shaking at 120 rpm. The proteins were denatured in 2× Laemmli buffer, heat denatured at 95 °C for 5 min and then analyzed by immunoblotting. For the identification of the PAP cleavage products following incubation with PaCP1, samples were separated by 12% SDS-PAGE and stained with Coomassie Brilliant Blue. The bands at 18 and 24 kDa were excised from the gel and subjected to an overnight trypsin digestion. The resulting peptide fragments from cleaved PAP were analyzed by the BioZone Mass Spectrometry Facility at the University of Toronto, Toronto, ON, Canada.

### 4.12. Immunoblotting

Equal amounts of protein were separated by 12% SDS-PAGE gel and transferred to nitrocellulose membranes. The membranes were blocked with either 5% milk in PBS-T or 5% BSA in TBS-T for 2 h, then incubated overnight at 4 °C with the appropriate primary antibody. After washing with PBS-T or TBS-T, the membrane was incubated with a horseradish peroxidase (HRP)-conjugated secondary antibody for 1.5 h. Proteins were detected on the membrane with enhanced chemiluminescent reagent kit (Thermo Fisher Scientific, Whitby, ON, Canada). The primary antibodies used were: polyclonal anti-PAP antibody (rabbit, 1:10,000), polyclonal anti-papain antibody (goat, 1:500; Cedarlane Labs, Burlington, ON, Canada, monoclonal anti-GFP antibody (mouse, 1:1000; Invitrogen, Burlington, ON, Canada), monoclonal anti-ribosomal protein S3 antibody (rabbit, 1:1000; Cell Signalling Technology, Whitby, ON, Canada), monoclonal anti-β-actin antibody (mouse, 1:1000; Novus Biologicals, Oakville, ON, Canada), and polyclonal anti-phospho-p44/42 MAPK antibody (rabbit, 1:1000; Cell Signalling Technology, Whitby, ON, Canada).

### 4.13. Peptide Treatment

To test the effect of the peptides derived from the 24 kDa PAP cleavage product on the phosphorylation of MAPK, six to eight leaf pokeweed plants were infiltrated with either water, 5 µM synthetic N-terminal peptide (purity > 80%, Millipore Sigma, Oakville, ON, Canada), 5 µM synthetic C-terminal peptide (purity > 80%, Millipore Sigma, Oakville, ON, Canada), and 5 µM synthetic flg22 (purity > 95%, PhytoTech Labs, Lenexa, KS, USA). After 24 h of infiltration, the infiltrated leaves were collected, and total proteins were isolated and analyzed by immunoblot as described above.

### 4.14. Isolation of Ribosomes and Depurination Assay

*N. benthamiana* ribosomes were isolated as previously described [71]. Ribosomes (50 µg) were incubated with either full-length PAP, the 24 kDa or 18 kDa PAP cleavage product (5 µg) for 30 min at 30 °C in RIP buffer (60 mM KCl, 10 mM Tris-HCl pH 7.4, 10 mM MgCl_2_) in a final volume of 100 µL. Equal volume of 2× extraction buffer (240 mM NaCl, 50 mM Tris-HCl pH 8.8, 20 mM EDTA, 2% SDS) was added following the incubation. rRNA was extracted using phenol-chloroform and precipitated in 0.3 M NaOAc and ethanol. Depurination of rRNA was assessed using a qRT-PCR based assay as previously described [72] with *N. benthamiana* specific 25S rRNA primers for the target gene product (Supplementary Table S2). Graphpad prism v.10 was used for the statistical analysis.

## Data Availability

Data is contained within this article. Raw data are available on request.

## Supplementary Materials

The following supporting information can be downloaded at: https://www.mdpi.com/article/10.3390/plants14152441/s1, Supplementary Table S1: PAP interactors identified by mass spectrometry; Supplementary Table S2: Primer sequences used for cloning and qRT-PCR.

## Abbreviations

The following abbreviations are used in this manuscript:

AF	Apoplastic Fluid
AtXCP1	*Arabidopsis thaliana* xylem cysteine protease 1
MAPK	Mitogen activated protein kinase
PaCP1	*Phytolacca americana* cysteine protease 1
PAP	Pokeweed antiviral protein
PLCP	Papain-like cysteine protease
RFU	Relative fluorescence units
RIP	Ribosome inactivating protein
