# Supplementary material for: A Novel Cysteine Protease from Phytolacca americana Cleaves Pokeweed Antiviral Protein Generating Bioactive Fragments
Source: Plants (Basel). 2025 Aug 7;14(15):2441. doi: 10.3390/plants14152441 (PMC12349160; doi:10.3390/plants14152441)
Supplement: Supplementary file 1 [file plants-14-02441-s001.zip › plants-3708013-supplementary.pdf]

Supplementary Table S1. PAP interactors identified by mass spectrometry.

Pokeweed Gene ID	Homologous Protein Name	Subcellular localization
anno2.g37031	Fructose-bisphosphate aldolase 1 (FBA1)	Chloroplast
anno1.g30684	Carbonic anhydrase (CA)	Chloroplast
anno2.g13520	Eukaryotic elongation factor 1-alpha (eEF1a)	Cytoplasm
anno1.g13250	Glyceraldehyde-3-phosphate dehydrogenase B (GAPB)	Chloroplast
anno1.g16742	Xylem cysteine protease (XCP1)	Extracellular space, vacuole
anno1.g27098	40S ribosomal protein S26 (RPS26)	Cytoplasm

Supplementary Table S2. Primer sequences used for cloning.

Name	Sequence	Used for
attB1 adapter	GGGGACAAGTTTGTACAAAAAAGCAGGCT	Full attB PCR
attB2 adapter	GGGGACCACTTTGTACAAGAAAGCTGGGT	Full attB PCR
attB1 For PAP-1	AAAAAGCAGGCTGTGAATACAATCATCTACAATGTTGGAAGTACCAC	Mature PAP Y2H
attB2 Rev PAP-1	AGAAAGCTGGGTTCATCAATAAGTTGTCTGACAGC	Mature PAP Y2H
attB1 For Pro-PaCP1	AAAAAGCAGGCTCTCGTGACTTCTCAATCGTCGGTTATTC	Pro-PaCP1 Y2H
attB1 For mPaCP1	AAAAAGCAGGCTCTAAGTCTGTAGACTGGAGAAAGA	Mature PaCP1 Y2H
attB2 Rev PaCP1	AGAAAGCTGGGTTCATCATCATTTCTTCTTGATAGG	Mature/Pro-PaCP1 Y2H
attB1 For Full PaCP1	AAAAAGCAGGCTCTATGGCTCTTTCCCTATACAACTT	PaCP1 agroinfiltration
attB2 Rev Full PaCP1	AGAAAGCTGGGTTTTTCTTCTTGATAGGGTAAGA	PaCP1 agroinfiltration
25S rRNA target For	TTACCCTACTGATGCCCGCGTC	qPCR depurination
25S rRNA target Rev	TGGCTTTTCAACCAAGCGCGATG	qPCR depurination
PaCP1 EcoRI For	CTCGGAATTCAAGTCTGTAGACTGGAGAAAGA	PaCP1 into pET28a
PaCP1 Blp1 Rev	CTCGGCTCAGCTCATCATTTCTTCTTGATAGG	PaCP1 into pET28a
NcoI N-PAP 24	ATATCCATGGAGTACGCCACTTTTCTGAATGATC	24 kDa PAP into pET28a
NcoI N-PAP 18	ATATCCATGGAGCGAAACAATTTGTATGTGAT	18 kDa PAP into pET28a
NotI C-PAP 24/18	ATATGCGGCCGCTCTCAACACTATCCACTTGGCA	24/18 kDa PAP into pET28a
